# Application of natural language processing to predict final recommendation of Brazilian health technology assessment reports

**DOI:** 10.1017/S0266462324000163

**Published:** 2024-04-12

**Authors:** Marilia Mastrocolla de Almeida Cardoso, Juliana Machado-Rugolo, Lehana Thabane, Naila Camila da Rocha, Abner Mácula Pacheco Barbosa, Denis Satoshi Komoda, Juliana Tereza Coneglian de Almeida, Daniel da Silva Pereira Curado, Silke Anna Theresa Weber, Luis Gustavo Modelli de Andrade

**Affiliations:** 1 Health Technology Assessment Unit, Hospital das Clínicas da Faculdade de Medicina de Botucatu, Botucatu, Brazil; 2 Laboratory of Data Science and Predictive Analysis in Health, Hospital das Clínicas da Faculdade de Medicina de Botucatu, Botucatu, Brazil; 3 Department of Health Research Methods, Evidence, and Impact, McMaster University, Hamilton, ON, Canada; 4 Biostatistics Unit, St Joseph’s Healthcare Hamilton, Hamilton, ON, Canada; 5 Faculty of Health Sciences, University of Johannesburg, Johannesburg, South Africa; 6 Department of Ophthalmology, Otorhinolaryngology and Head and Neck Surgery, Medical School (FMB) of São Paulo State University, Botucatu, Brazil; 7 Department of Collective Health, University of Campinas, Campinas, Brazil; 8 Department of Management and Incorporation of Health Technologies, Ministry of Health, Brasilia, Distrito Federal, Brazil; 9 Department of Internal Medicine, Medical School (FMB) of São Paulo State University, Botucatu, Brazil

**Keywords:** health technology assessment, Brazil, prediction analysis, machine learning, natural language process

## Abstract

**Introduction:**

Health technology assessment (HTA) plays a vital role in healthcare decision-making globally, necessitating the identification of key factors impacting evaluation outcomes due to the significant workload faced by HTA agencies.

**Objectives:**

The aim of this study was to predict the approval status of evaluations conducted by the Brazilian Committee for Health Technology Incorporation (CONITEC) using natural language processing (NLP).

**Methods:**

Data encompassing CONITEC’s official report summaries from 2012 to 2022. Textual data was tokenized for NLP analysis. Least Absolute Shrinkage and Selection Operator, logistic regression, support vector machine, random forest, neural network, and extreme gradient boosting (XGBoost), were evaluated for accuracy, area under the receiver operating characteristic curve (ROC AUC) score, precision, and recall. Cluster analysis using the k-modes algorithm categorized entries into two clusters (approved, rejected).

**Results:**

The neural network model exhibited the highest accuracy metrics (precision at 0.815, accuracy at 0.769, ROC AUC at 0.871, and recall at 0.746), followed by XGBoost model. The lexical analysis uncovered linguistic markers, like references to international HTA agencies’ experiences and government as demandant, potentially influencing CONITEC’s decisions. Cluster and XGBoost analyses emphasized that approved evaluations mainly concerned drug assessments, often government-initiated, while non-approved ones frequently evaluated drugs, with the industry as the requester.

**Conclusions:**

NLP model can predict health technology incorporation outcomes, opening avenues for future research using HTA reports from other agencies. This model has the potential to enhance HTA system efficiency by offering initial insights and decision-making criteria, thereby benefiting healthcare experts.

## Introduction

Health demands constantly grow and evolve to meet the emerging and changing needs of society in response to social, political, economic, and technological changes. The health technology assessment (HTA) process has been increasingly applied throughout the world as the main method to inform policy and decision-making in healthcare on different types of technologies such as medical-hospital materials, equipment, drugs, and procedures; besides, the HTA approach can give information about organizational or educational systems, care programs and protocols (1).

Over the years, several HTA agencies have been created in different countries with the purpose of improving decision-making regarding investments and disinvestments in health. These HTA processes have been the object of several studies to compare differences and similarities between them (e.g., criteria, health priorities, challenges or limitations, among other aspects) (2–4). Heupink et al. (5) highlight that the HTA process requires time and resources. Because of this, the application of the reusing process of existing HTAs can be useful, particularly for critical policy decisions. The author called this process as transferring existing HTAs between different contexts (i.e., countries, regions, or HTA agencies). The benefits could be to accelerate the production of HTA’s reports, reduce duplication, improve knowledge sharing, and subsidy countries with fewer resources for HTA (5).

Still, in the field of HTA, researchers have studied implicit factors involved in the deliberative process. A systematic review published in 2022 identified studies that categorized these factors in different countries. The HTA deliberative process is influenced by implicit factors related to the behavior and personal values of the individuals involved as well as to the context in which this process is performed. According to the authors, personal biases had consequences in resource allocation which compromise the fairness and legitimacy of HTA decisions. To tackle these hazards, some HTA agencies have been using frameworks for deliberative processes. However, according to the authors, inconsistencies, variability, and lack of predictability have been reported in current HTA value frameworks (6).

The quality of HTA reports has been increasingly gaining the attention of researchers (7–11). The World Health Organization conducted two global surveys in 2015 and 2020/2021 about HTA processes (12). Among the responses from 127 countries, data related to the recommendation process showed if, and how organizations share HTA information. Almost 50 percent of respondents answered that reports and recommendations are the most likely materials to be published and publicly available (12).

These surveys highlight the importance of understanding how HTA reports are developed, as these are the main formats to share information (e.g., evidence synthesis, economic evaluation), and it’s the main document used by HTA agencies to get the information to support decision-making.

According to Demner-Fushman et al. (13), the narrative text is an important component of communication in health care, which includes, besides patient-accessible information in health reports, general biomedical knowledge papers, information from textbooks, and web resources. For Harrison and Sidey‑Gibbons (14), medical records, patient feedback, assessments of doctors’ performance, and social media comments are examples of data that support clinical decision-making and quality improvement.

Natural language processing (NLP) is an artificial intelligence (AI) technique that has been used to analyze narrative/written text in a variety of fields such as environment, governance, health, education, and finance. NLP techniques receive complex text as input, and can interpret data as outputs, after automated processing of the original data (14;15).

Some initiatives have been exploring studies that discuss NLP as a means to predict health outcomes (16;17). A scoping review mapped AI methods used to generate outcome data to provide decision-makers with knowledge and expand perspectives for HTA. NLP represented 1.4 percent of all methods and was predominantly used to predict the following outcomes: safety, efficacy/effectiveness, and morbidity. Considering the type of technology under assessment, NLP has been applied to evaluate aspects related to drugs and medical devices (18).

As an example of the applicability of NLP in the prediction of outcomes, one could mention the judicial field, in which NLP has been applied to identify facts, relationships, and affirmation in the textual data. Additionally, NLP contributed to the time and cost reduction of judicial services (19). Furthermore, it could unveil data patterns driving decisions with high accuracy (20–24). Alcántara Francia et al. (19) identified several advantages of this application such as the reduction of case complexity. Complementary, courts can use Al to expand the workforce to achieve higher productivity with limited resources. Not only that, but the authors also mentioned the use of AI could potentially reduce human bias and guarantee impartiality (19).

In considering all the points discussed above, this study aimed to build an NLP model to predict the HTA final decisions. Our main hypothesis is that based on the semantic analysis of summaries from HTA reports, this model could potentially improve the throughput of HTA systems by supporting decision-makers with a set of features or elements that could indicate odds of approval. As a pre-analytical step, it could identify technologies according to the public health system´s priorities compare patterns in earlier decisions with the same characteristics or even reduce the time of the process.

## Method

In studies that applied NLP in the judicial system, different models that differ in terms of method and accuracy are presented and compared (19, 21, 24). In this sense, it was decided to carry out an analysis applying different prediction models such as Least Absolute Shrinkage and Selection Operator (LASSO), logistic regression, support vector machine (SVM), random forest, neural network, and extreme gradient boosting (XGBoost) in order to verify which one has the greatest capacity to process the data of interest with greater accuracy, area under the receiver operating characteristic curve (ROC AUC), precision and recall.

### Data source

Data were obtained from the official website of the Brazilian National Committee for Health Technology Incorporation (CONITEC), including all summaries of official recommendation reports conducted between 2012 and 2022, (https://www.gov.br/conitec/pt-br/assuntos/avaliacao-de-tecnologias-em-saude/recomendacoes-da-conitec).

On the website of the CONITEC, since 2012, all HTA documents are available for public access, which include updated guidelines, HTA recommendation reports, and new guidelines. For this study, were considered all HTA recommendations reports because these documents precede all updated guidelines, new guidelines, and present all information to support decision-making (economic evaluation, budget impact, clinical evidence, public consultation, and preliminary and final CONITEC´s decision). There is a template for this HTA recommendation report. However, since 2012, this template has been modified and one of these modifications was the inclusion of a summary where all information about all topics mentioned above is included. The initial idea for this first experiment is to apply the model only in this summary. The CONITEC is a permanent committee of the Ministry of Health, with the purpose of advising the Federal Government in the attributions related to the incorporation, exclusion, or alteration of health technologies, as well as in the constitution or alteration of clinical protocols and therapeutic guidelines at the public health system (SUS). This Committee was created by Law 12,401, of 28 April 2011, and from 2012 the HTA documents analyzed were available for public access.

The analysis was developed using Jupyter Notebook and Python 3.9.12.

### Data preparation

#### Definition of sample and outcome

All reports that met the following criteria were considered for analysis: (1) were classified as a recommendation report; (2) presented a report summary; and (3) presented a final result “incorporated” or “not incorporated.”

For each synthesis the following data were analyzed: (i) type of requester/demandant (government, industry, or society); (ii) type of technology (drug, procedure, product); (iii) data on evidence synthesis; (iv) data on recommendations by other agencies; (v) data on technological horizon scanning; (vi) data on patients’ perspectives and; and (vii) general/final considerations.

The report’s synthesis contains a section describing the preliminary and final recommendation by CONITEC. Sections regarding these decisions were excluded from the analyzed text. Documents published as clinical guidelines were not eligible for this study.

#### Text normalization

Text fields were preprocessed using an automated form by cleaning the text data by removing any irrelevant information such as stop words (e.g., articles, adverbs, prepositions, and conjunctions), punctuation marks, and accents. Stop words are those that are frequently used in a language but do not carry any meaning on their own (e.g., in English “the,” “a/an,” in Portuguese, “o/a/os/as,” and “um/uma/uns/umas,” respectively). Removing them helps to reduce the dimensionality of the dataset and to improve the accuracy of the model. Punctuation marks and accents were also removed to standardize the text data. Finally, all text data were converted to lowercase to ensure consistency in the data.

Stemming is another technique in data preprocessing performed in an automated way. It shortens words to their base or root form, for example, derived words such as “running,” “run,” and “runner” can be reduced to their base form “run” decreasing the dimensionality of the dataset. This technique improves the accuracy of the model by reducing the number of features.

#### Text vectorization

Feature extraction involves converting the text data into numerical values that can be used in machine learning algorithms. The most commonly used method for feature extraction in NLP is the Term Frequency-Inverse Document Frequency (TF-IDF) method. TF-IDF is a statistical approach that gives weights to words in a document based on their frequency and importance. The TF-IDF score is higher for words that are crucial but rare in the document. Therefore, a term’s TF-IDF score is more significant if it appears frequently in the document but seldom in the corpus. This indicates the term’s importance for that specific document and its potential to differentiate it from other documents in the collection (25).

Another technique of feature extraction is n-grams, which involves capturing the context of the text (26). n-Grams are sequences of “n” words, and by considering “n” as 2 or 3, we can capture the context of the text. They can capture the frequency and co-occurrence of words in a text, as well as their order and context. For instance, in the sentence “Conduct market research before launch,” the 2-grams (also known as bigrams) are “Conduct market,” “market research,” “research before,” and “before launch.”

After preprocessing, tokenization of the summary field was performed and the NLP techniques TF-IDF and n-grams were applied for different data sizes. Tokenization is the process of breaking down a text into smaller units called tokens. These tokens are typically words, phrases, or symbols, and they serve as the building blocks for various NLP tasks. Tokenization is a crucial step in language processing pipelines, enabling computers to analyze and understand textual data more effectively (27;28).

#### Train/test split

To evaluate the performance of the model, the preprocessed dataset was randomly split into a training set (75 percent of the reports, n = 324) and a testing set (25 percent of the reports, n = 108) using sci-kit-learn. The training set is used to train the model, and the testing set is used to evaluate the performance of the model on unseen data. Additionally, tenfold cross-validation was applied during the training process to ensure robustness and reliability in assessing the model’s generalization capabilities.

### Modeling

#### Model type

In this step, we evaluate several classification models to predict the label of the text data. We started by using LASSO regression with an alpha value of 0.01 to reduce the noise in the data and improve the accuracy of the model. LASSO regression is a linear model that uses L1 regularization to shrink the less important features to zero (29).

In sequence, logistic regression and SVM models were applied. Logistic regression is a linear model used for binary classification, and SVM is a nonlinear model used for both binary and multi-class classification (30;31). Additionally, a random forest classifier was used, which is an ensemble learning method that utilizes decision trees. Random forests can capture the complex interactions between features and are known for their high accuracy (32).

Deep learning involves building Neural Networks with multiple layers to improve the accuracy of the model. In this study, Keras and Tensorflow were used to build a neural network with ten epochs, a batch size of thirty-two, and a validation split of 0.1. A binary cross-entropy loss function and the Adam optimizer for training the neural network were used. Lastly, the XGBoost algorithm, which is an ensemble learning method that utilizes decision trees was applied (33). XGBoost is known for its high accuracy and is widely used in NLP applications. The max depth to 5 and the learning rate of 0.1 was set. The optimal hyperparameters for the model were selected using tenfold cross-validation resampling techniques with the aim of maximizing the area under the ROC curve.

##### Model selection

Each model on the preprocessed data was trained and its performance was evaluated using various metrics, such as accuracy, precision, recall, and the ROC AUC. Based on these metrics, the best-performing model was selected for further analysis.

The accuracy indicates the overall performance of the model of all the classifications, and how many did the models classified correctly. The precision is a metric that evaluates the number of true positives over the sum of all positive values. Recall (Sensitivity) evaluates the ability of the method to successfully detect results classified as positive (34)

The ROC AUC is a graph that allows you to evaluate a binary classifier. This visualization takes into account the rate of true positives (sensitivity) and the rate of false positives (specificity), in other words, the rate of “approved” predicted by the model that was recommended in the report, and the rate of “rejected” according to the model, and not recommended in the report. This graph allows you to compare different classifiers and define which one is better based on different cut-off points.

##### K-mode clustering analysis

Once we had extracted the features from the text data, we performed a *k*-mode clustering analysis. *K*-mode clustering is a variant of *k*-means clustering that is designed to work with categorical data, such as text data (35;36).

We used the elbow curve method to determine the optimal number of clusters to use in our analysis. The elbow curve plots the sum of squared errors (SSE) for each number of clusters, and we selected the point where the SSE begins to level off (the “elbow” of the curve) as the optimal number of clusters to use. Based on this analysis, we built two clusters.

##### Model evaluation

Finally, we evaluated the performance of the selected model by comparing its accuracy *to t*he accuracy of the other models. We also analyzed the results of the *k*-mode clustering analysis to identify any patterns or trends within each cluster.

Overall, this methodology allowed us to identify patterns and trends in the text data and build a model that accurately predicted the recommendation outcome.

#### Interpretability

The interpretability of the model is a critical aspect, and XGBoost incorporates strategies such as SHAP (SHapley Additive exPlanations) plots to facilitate model explanation (Figure 2). The SHAP is used to generate interpretable explanations for individual predictions. By highlighting the features that significantly influence the model’s decision, this approach makes the decision process more understandable. The SHAP offers a distinct advantage over more sophisticated NLP models in terms of providing transparent insights into the model’s decision-making process. The simplicity and clarity offered by SHAP plots can be particularly beneficial for understanding and communicating the model’s behavior, making it a valuable tool in scenarios where interpretability is paramount.

## Results

During the period from 2012 to 2022, 627 reports were published on the CONITEC website, after considering the exclusion criteria, 432 remained for the final analysis (Supplementary Figure S1. Flow Diagram adapted from Page et al., 2020 (37)).

### Model statistics

The neural network model demonstrated the best accuracy metrics with a precision of 0.815, accuracy of 0.769, ROC AUC of 0.871, and a recall of 0.746, followed by the XGBoost model with a precision of 0.745, accuracy of 0.704, ROC AUC of 0.811, and a recall of 0.695. Table 1 presents the accuracy metrics of all models in the test set.

**Table 1.** Performance (accuracy, ROC AUC, precision, and recall) of each model, Brazil, 2023

	Accuracy	ROC AUC	Precision	Recall
LASSO	0.639	0.672	0.727	0.542
	(0.546–0.731)	(0.560–0.741)	(0.591–0.860)	(0.415–0.672)
Logistic regression	0.704	0.710	0.776	0.644
	(0.611–0.787)	(0.620–0.792)	(0.645–0.889)	(0.521–0.762)
SVM	0.750	0.759	0.848	0.661
	(0.676–0.833)	(0.685–0.833)	(0.733–0.942)	(0.550–0.778)
Random forest	0.630	0.594	0.598	0.983
	(0.537–0.722)	(0.538–0.655)	(0.490–0.694)	(0.945–1.000)
Neural network	0.769	0.871	0.815	0.746
	(0.630–0.880)	(0.649–0.880)	(0.667–0.978)	(0.517–0.879)
XGBoost	0.704	0.811	0.745	0.695
	(0.583–0.843)	(0.583–0.851)	(0.592–0.920)	(0.516–0.847)

LASSO, least absolute shrinkage and selection operator; ROC AUC, area under the receiver operating characteristic curve; SVM, support vector machine; XGBoost, extreme gradient boosting.

**Figure 1.**
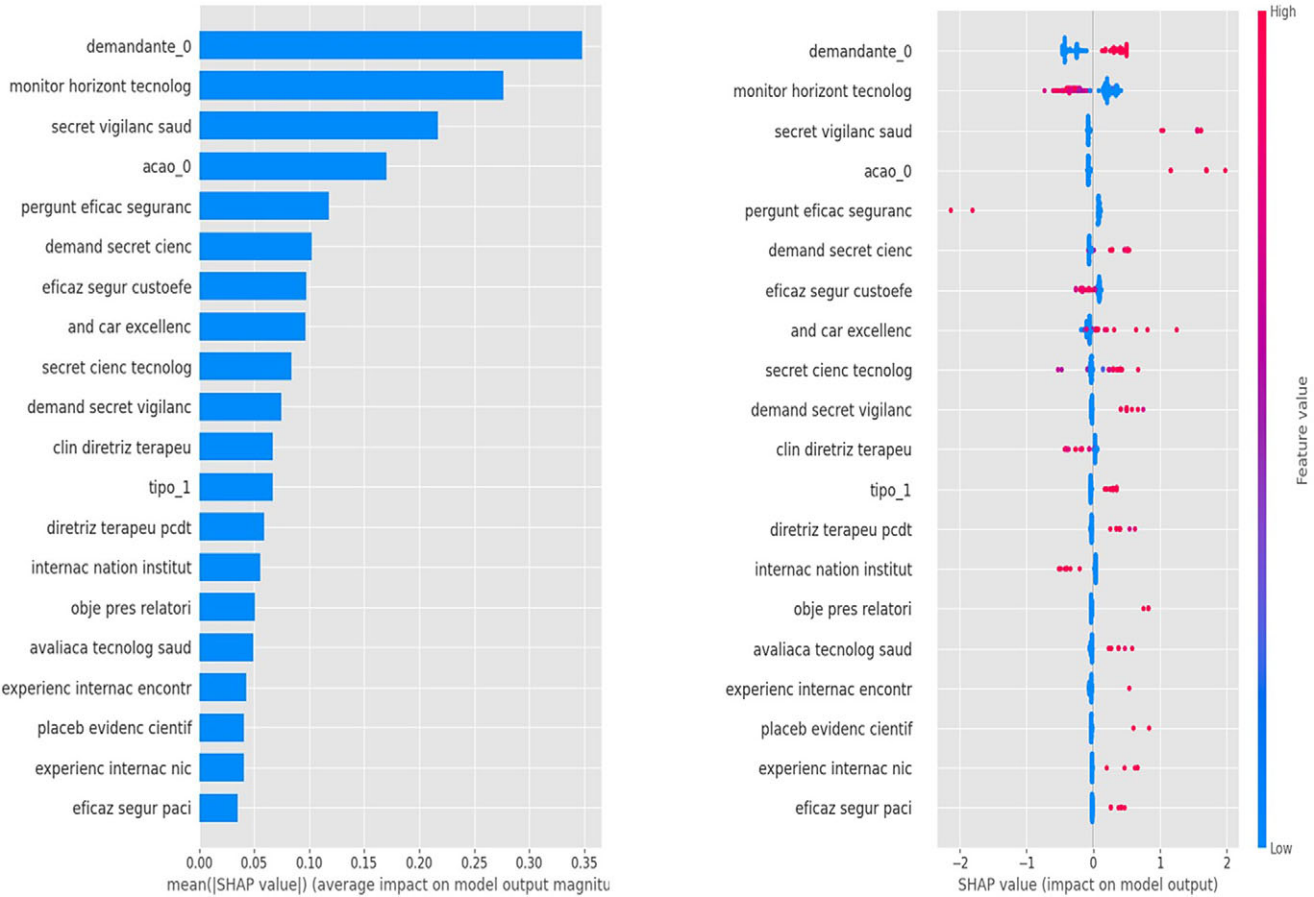
The top twenty important features are determined by SHAP values for the XGBoost model. The mean absolute SHAP values on the left side show global feature importance, while the local explanation summary on the right side indicates the relationship between a variable and the process outcome. Positive SHAP values indicate approval, while negative values indicate non-approval.

According to the XGBoost model, the variables with the stronger influence on the committee’s decision are presented in Figure 1.

It is possible to observe that the variables related to government (*“demandante_0*,” *“secret vigilanc saud*,” *“demand secret cienc*,” *“secret cienc tecnolog*,” *“demand secret vigilanc”*) had a great weight in the final decision. Also, variables related to international experience (*“internac nation institut*,” *“experienc internac encontr*,” *“experienc intern nic*,” *“and car excellence”*) had either a positive or negative weight for incorporation.

Regarding the type of action, the reports that propose to expand the use of technology had a greater weight for the incorporation (*“acao_0”*). Likewise, procedural technology, even less frequently presented when compared to drugs, had a greater weight for the incorporation (*“tipo_1”).*

### Cluster analysis

In the cluster analysis, the *k*-modes clustering algorithm to further investigate the results. Based on the elbow method, two clusters were selected, one was the “recommended technology group” being labeled as “0” and the second one, the “non-recommended technology group” being labeled as “1.”

**Figure 2.**
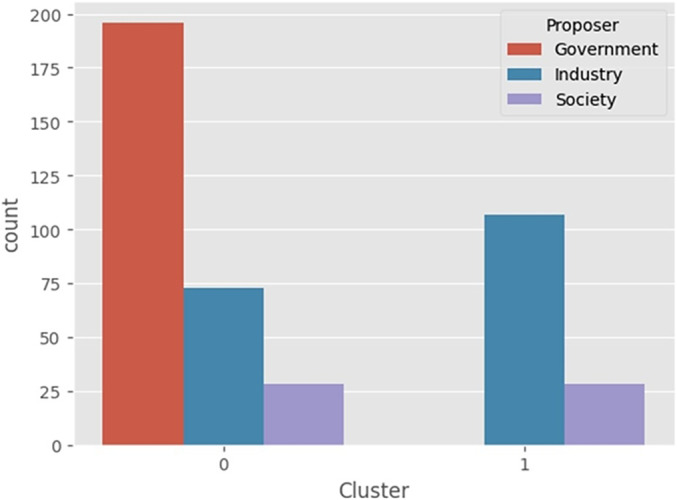
Composition of groups 0 and 1 by proponent: government, industry, and society.

**Figure 3.**
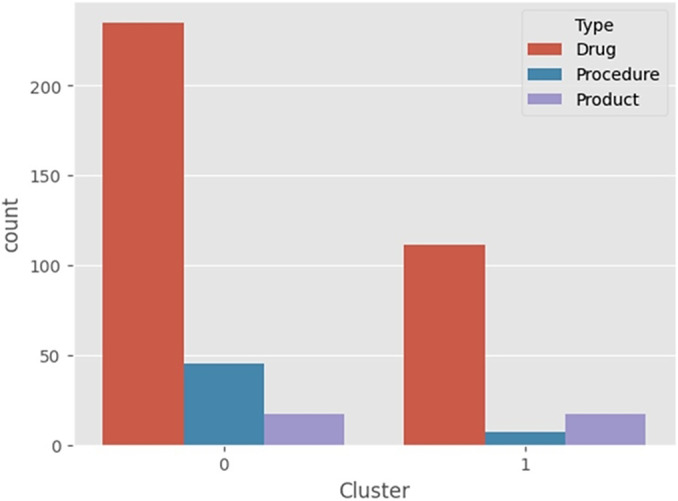
Composition of groups 0 and 1 by type: drugs, procedures, and products.

In both analyses, presented in Figures 2 and 3, the clustering algorithm, and the top twenty features by importance from SHAP Value (XGBoost model) showed similar results. Approved technologies were predominantly composed of drug analysis, with the government as the main requester. In contrast, for those not approved, most were drug analysis, but the industry was the main requester.

**Figure 4.**
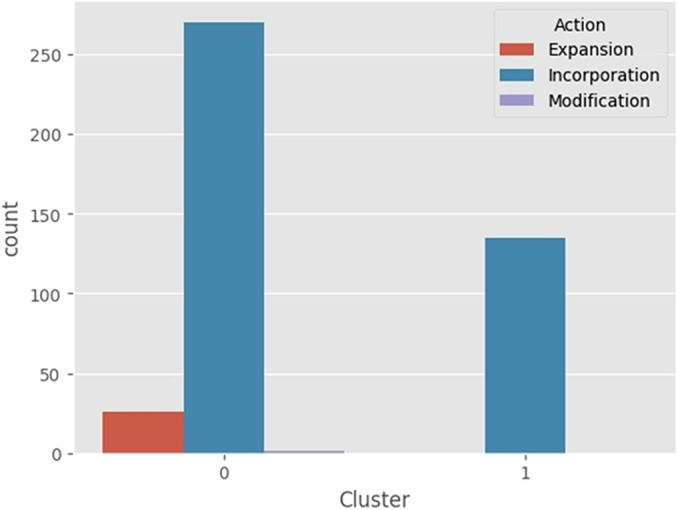
Composition of groups 0 and 1 by action: expansion, incorporation, and modification.

Similarly, technology involving “procedure,” also classified in group 0 and ranked 12th in SHAP values, has a higher chance of approval than “Drug” and “Product” projects as presented in Figure 4.

Projects that involve expansion have been classified into group 0, which primarily includes approved projects. Furthermore, based on the SHAP values in the XGBoost model, expansion projects have been identified as the fourth most important feature. As a result, these projects have a higher likelihood of approval when compared to those involving incorporation or modification.

## Discussion and implementation consideration

According to the Brazilian legislation for the incorporation of technologies in the SUS, several criteria must be in the evaluations, namely: scientific evidence on the efficacy, accuracy, effectiveness, and safety of the drugs, product, or procedure under analysis. Also, a comparative economic evaluation must show the benefits and costs concerning the technologies already incorporated into the SUS, as well as, the budget impact of incorporation into the health system. However, Souza et al. (38) found that despite not being explicit in the legislation, in practice there are other criteria that are taken into account in the decision process, depending on the rapporteur or the opinion of the members of the commission.

The result of this study showed that some characteristics of the reports (type of technology and the requester’s profile) predict the incorporation, and are not directly related to the criteria settled down. Previous studies that characterized the CONITEC by other methods, in particular descriptive analysis, showed a similar profile, with the drug technology being the most evaluated and approved, and the “government” claimant having the highest number of approved technologies (4;9;39).

Another aspect identified by the model was the presence of tokens with greater weight associated with a favorable outcome for technology recommendation, which is related to the international experiences of other agencies.

Brazilian researchers have compared Brazilian processes with international agencies since the selected countries are relevant to the incorporation processes, due to the consolidation time and because they have contexts of universal health systems. The study by Lima SGG et al. (7) analyzed the technology incorporation process in Brazil, Australia, Canada, and the UK. One of the key topics of their discussion is the process of (pre)selection and/or prioritization of themes to be analyzed by the agencies, adopted by all countries, except CONITEC. According to the survey, the countries have defined criteria for a pre-selection: alternative technologies; budget impact; clinical impact; controversial nature of proposed technology; disease burden; economic impact; ethical, legal, and psychosocial implications; availability and relevance of evidence; level of interest (government, health professionals and patients); review opportunity; variation in the utilization rate.

Still, at the international level, researchers have also sought to know implicit factors in the deliberative processes in HTA. In 2022, a systematic review identified and categorized these factors for the deliberative process of drugs in Germany, France, Italy, the UK, and Spain. According to the authors, the HTA deliberative process is influenced by implicit factors related to the behavior and personal values of the involved individuals, as well as the context in which this process is performed. They characterized the categories based on the frequency mentioned in the literature: ethics, psychology, qualification and experience, politics and society, culture, functional role, and disease perception. Ethical issues were the most frequent category, especially value judgments. Psychologies (personality and subjective), culture, functional role, qualification and experience, and disease perception were factors that were related to who provided the recommendations. Political (process, pressure, and influence), and organizational culture were also observed. According to the authors, personal biases had consequences on resource allocation compromising the fairness and legitimacy of HTA decisions. To handle these aspects, some HTA agencies have used frameworks for specific deliberative processes. However, according to the authors, inconsistencies, variability, and lack of predictability have been reported in the current HTA value frameworks (6).

For Furtado and Lassance (40), taking a new decision, or maintaining (or not) the same in public policy, often involves questions of criticality, urgency, dimension, multi-causality, political-institutional multiplicity, and implementation deadline limitations. For the authors, computational models contribute to evaluation structures through inductive and deductive processes based on algorithms. These methods can detail the results, thus allowing the interlocutors previously to understand the effects and to participate in the decision-making process. Through simulations, it is possible to reduce uncertainties, as well as, to identify measures and anticipate other possibilities. Similarly, NLP is inserted in the judicial systems in several countries for the screening of outcomes prediction, helping in the simulation of an appeal, and dropping the number of unsuccessful ones (21).

Enhance the workflow of the courts by automatically evaluating an appeal and suggesting the best outcome, creating automated management systems for the most common cases. This technology could potentially improve the throughput of legal systems by supporting federal judges and their staff (24).

This study confirmed that an IA model of semantic analysis of the HTA process can predict the CONITEC recommendation outcome, which means that specific characteristics will influence the decision. These results enhance the necessity of reflection for the creation of new structures that potentially might improve the throughput of the HTA system by supporting experts’ decision-making in a pre-selection step.

However, in addressing the concern about the possibility of misalignment between the classifier’s predictions and the decisions of the review committee, the present approach is centered on enhancing the interpretability and transparency of the model to foster trust and facilitate seamless integration of the classifier into the decision-making workflow.

Thus, some strategies are proposed in the following sections.

### Model documentation

This documentation will serve as a comprehensive resource for the review committee, offering insights into the model’s inner workings and its learning process. This transparency is crucial for ensuring that decision-makers can confidently assess and understand the model’s capabilities and limitations.

### User-friendly interface

A user-friendly interface could be designed that allows decision-makers to interact with the model easily. This interface could provide not only the final predictions but also relevant information on the features contributing to each prediction. A clear and intuitive presentation of the model’s output can significantly enhance interpretability.

### Continuous collaboration with decision-makers

Collaboration with the review committee could be ongoing, involving regular meetings to address any concerns, provide clarifications, and receive feedback. This iterative process ensures that decision-makers are actively involved in the deployment and evolution of the model, fostering a sense of ownership and confidence in its utility.

#### Education and training

A training session could be offered to familiarize decision-makers with the underlying concepts of the model and its application. This educational initiative aims to empower the committee with the knowledge necessary to interpret and critically assess the mode’s predictions.

By implementing these strategies, the potential confusion and obstacles in the decision-making process can be minimized, ensuring that the classifier serves as a valuable tool that aids, rather than complicates, the HTA.

It is important to highlight that in 2023, a new HTA process was implemented in Brazil, in which the CONITEC is no longer composed of a single group of members, but of three thematic committees (drugs, guidelines, or products and procedures). Therefore, in this scenario, models such as the one proposed can facilitate the decision-making process.

## Limitations

The analysis was made in the text of decisions from synthesis and not from all reports. Although the accuracy was with a value considered adequate, it is believed that there are possibly other factors that have greater weight in decision-making and that were excluded from the synthesis, especially those related to the numerical values contained in the economic and budget impact analyses. The other limitation is not having HTA studies with similar analyses to compare.

The fact that there is no computerized system for including HTA report data, as is the case in the judicial system, was also considered a limitation. Therefore, for the model to be applied throughout the report, it would be important to analyze computerized forms of application of the evaluation processes. Once computerized, in addition to the analysis performed, it would be possible to identify other data of interest through the creation of fields established according to criteria. For example, data that represent importance for the patient, and priority for the health system, among others.

The last limitation to point out is the use of simpler NLP models, as compared to complex ones. Tokenization, n-grams, and TF-IDF are considered simpler because they operate on more basic linguistic principles, but have a clear and more interpretable structure, while GPT models, besides powerful, are more complex and involve intricate neural network architectures, making them harder to interpret and understand. The choice between these approaches often depends on the specific task and the available resources. Further studies are suggested to explore other more complex models.

## Conclusion

This study presented an NLP model for the identification of possible predictors for the final decision process on the incorporation of health technologies in the Brazilian Public Health System (SUS), opening paths for future work using HTA reports coming from other HTA agencies. This model could potentially improve the throughput of HTA systems by supporting experts given some prediction/factors/criteria for approval or not as a previous pre-section step.

In addition, this model could be validated and replicated in other similar contexts using the complete report from HTA agencies. However, considering the different application systems in different countries, it will be necessary to adequate the model to the HTA process in each context.

In this sense, one of the challenges for the model proposed in this study is to adjust it based on what you want to extract from the unknown data of the new database and make reliable predictions. Another challenge is to think of this model as a proposal that is not restricted to just the known base but has the ability to generalize to bases that differ from the one used in training.

## Supporting information

Cardoso et al. supplementary material Cardoso et al. supplementary material

## Supplementary material

The supplementary material for this article can be found at http://doi.org/10.1017/S0266462324000163.

## Key messages

1. What was known before this study?
  - Considering the high volume of submissions processed by health technology assessment (HTA) agencies, an automated approach based on artificial intelligence to determine key factors associated with timely evaluation approval would be beneficial.
  - The HTA deliberative process could be influenced by implicit factors related to behavior aspects and personal values of individuals involved as well as to the context in which this process is performed. To handle these aspects, some HTA agencies have been using frameworks for specific deliberative processes. However, inconsistencies, variability, and lack of predictability have been reported in current HTA value frameworks.
2. What are the key findings from the study?
  - Some tokenization identified that linguistic markers could potentially contribute to the prediction of incorporation decisions by the Brazilian HTA Committee, such as quotes about international HTA agencies’ experience and the government as the main requester (demandant).
  - A cluster analysis was conducted, revealing the existence of two primary groups. The first group consists mainly of drug evaluations, which were primarily requested by the government and had a high approval rate for most projects. The second group, which also evaluated drugs, was mostly composed of industry applicants and had a low approval rate for most projects. Some similarities were found between the extreme gradient boosting and cluster analysis results, such as the government being the primary “demandant,” expansion as the primary “action,” and procedure as the primary “type” for approved projects.
3. What are the implications of the findings?
  - The use of natural language processing allowed the identification of predictive features within the Brazilian HTA Committee associated with the final decision process on health technology incorporation.
  - Artificial intelligence could be used as an auxiliary to the early identification of factors that could indicate odds of approval or, as a pre-analytical step, it could identify technologies according to the public health system´s priorities or compare patterns in earlier decisions with the same characteristic or even reduce the time of the process.

## References

[1] Bertram M, Dhaene G, Edejer TTT, editors. Institutionalizing health technology assessment mechanisms: A how to guide. Geneva: World Health Organization; 2021.

[2] Zisis K, Naoum P, Athanasakis K. Qualitative comparative analysis of health economic evaluation guidelines for health technology assessment in European countries. Int J Technol Assessment Health Care. 2021;37(e2):1–8. 10.1017/S0266462320002081 33298238

[3] Pereira VC. Framework de suporte à tomada de decisão no processo de reavaliação das tecnologias em saúde pela Conitec, Tese (Doutorado em Ciências da Saúde) na Universidade de Brasília, UNB; 2018.

[4] Elias FTS, Morais RDGM, Silva ET, Pereira DCR. Studies of the Brazilian Network for Health Technology Assessment (Rebrats) of 2004‑2015. Com Ciências Saúde. 2016;27(1):53–70.

[5] Heupink LF, Peacocke EF, Sæterdal I, Chola L, Frønsdal K. Considerations for transferability of health technology assessments: A scoping review of tools, methods, and practices. Int J Technol Assessment Health Care. 2022;38(1):e78. 10.1017/S026646232200321X 36321421

[6] Monleón C, Späth HM, Crespo C, Dussart C, Toumi M. Systematic literature review on the implicit factors influencing the HTA deliberative process in Europe. J Mark Access Health Policy. 2022;10(1):2094047. 35811835 10.1080/20016689.2022.2094047 PMC9267410

[7] Lima SGG, de Brito C, de Andrade CJC. Health technology assessment in Brazil – An international perspective. Ciência Saúde Coletiva. 2019;24(5):1709–1722. 31166506 10.1590/1413-81232018245.17582017

[8] Silva HP, Elias FTS. Incorporation of technologies by the Canadian and Brazilian health systems: Prospects for progress in assessment processes. Cad Saúde Pública. 2019;35(2):e00071518. 31432894 10.1590/0102-311X00071518

[9] Yuba TY, Novaes HMD, de Soárez PC. Challenges to decision-making processes in the national HTA agency in Brazil: Operational procedures, evidence use and recommendations. Health Res Policy Syst. 2018;16(1):40. 29751764 10.1186/s12961-018-0319-8 PMC5948855

[10] Pereira VC, Salomon FCR, Souza AB. Criteria for decison-making regarding health technology incorporation in Brazil and throughout the world. Rev Eletrôn Gestão Saúde. 2015;6(4):3066–3093.

[11] Cardoso M, Thabane L, Rugolo J, et al. PP43 impact of the COVID-19 pandemic in the Brazilian National Committee for Health Technology Incorporation (CONITEC) recommendation process. Int J Technol Assessment Health Care. 2022;38(S1):S55.

[12] World Health Organization (WHO). Health technology assessment survey 2020/2021 – Main findings. Cited 19 February 2023. Available from: https://www.who.int/data/stories/health-technology-assessment-a-visual-summary.

[13] Demner-Fushman D, Elhadad N, Friedman C. Natural language processing for health-related texts. In: Shortliffe EH, Cimino JJ, editors. Computer applications in health care and biomedicine. Cham: Springer; 2021. p. 241–272.

[14] Harrison CJ, Sidey-Gibbons CJ. Machine learning in medicine: A practical introduction to natural language processing. BMC Med Res Methodol. 2021;21:158. 34332525 10.1186/s12874-021-01347-1 PMC8325804

[15] Andrew C, Scott, AC, Solórzano JR, Moyer JD, Hughes BB. The future of artificial intelligence. Int J Artif Intell Mach Learn. 2022;2(1):1–37.

[16] Feder A, Keith KA, Manzoor E, et al. Causal inference in natural language processing: Estimation, prediction, interpretation and beyond. Trans Assoc Comput Linguist. 2022;10:1138–1158.

[17] Velupillai S, Suominen H, Liakata M, et al. Using clinical natural language processing for health outcomes research: Overview and actionable suggestions for future advances. J Biomed Inform. 2018;88:11–19. 30368002 10.1016/j.jbi.2018.10.005 PMC6986921

[18] Graili P, Ieraci L, Hosseinkhah N, Argent-Katwala M. Artificial intelligence in outcomes research: A systematic scoping review. Expert Rev Pharmacoecon Outcomes Res. 2021;21(4):601–623. 33554681 10.1080/14737167.2021.1886083

[19] Alcántara Francia OA, Nunez-del-Prado M, Alatrista-Salas H. Survey of text mining techniques applied to judicial decisions prediction. Appl Sci. 2022; 12(20):10200.

[20] Aletras N, Tsarapatsanis D, Preoţiuc-Pietro D, Lampos V. Predicting judicial decisions of the European court of human rights: A natural language processing perspective. PeerJ Comput Sci. 2016*;* 2:e93

[21] Menezes-Neto JE, Clementino MBM. Using deep learning to predict outcomes of legal appeals better than human experts: A study with data from Brazilian federal courts. PLoS One. 2022;17(7):e0272287. 35901178 10.1371/journal.pone.0272287 PMC9333285

[22] Coulthard B, Taylor BJ. Natural language processing to identify case factors in child protection court proceedings. Methodol Innovat. 2022;15(3):222–235.

[23] Sert MF, Yıldırım E, and Haşlak İ. Using artificial intelligence to predict decisions of the Turkish constitutional court. Soc Sci Comput Rev. 2022;40(6):1416–1435.

[24] Mumcuoğlu E, Öztürk CE, Ozaktas HM, Koç A. Natural language processing in law: Prediction of outcomes in the higher courts of Turkey. Inform Proc Management. 2021;58:102684.

[25] Sparck Jones K. A statistical interpretation of term specificity and its application in retrieval. J Document. 1972;28(1):11–21.

[26] Damashek M. Gauging similarity with n-grams: Language-independent categorization of text. Science. 1995;267(5199):843–848. 17813910 10.1126/science.267.5199.843

[27] da Rocha NC, Barbosa AM, Schnr YO, et al. Natural language processing to extract information from portuguese-language medical records. Data. 2022;8(1):11.

[28] Webster JJ, Kit C. Tokenization as the initial phase in NLP. In: COLING 1992, Volume 4: The 14th international conference on computational linguistics, 1992.

[29] Tibshirani R. Regression shrinkage and selection via the lasso. J R Statist Soc B Methodol. 1996;58(1):267–288.

[30] Vapnik V. The nature of statistical learning theory, 2nd ed. New York, NY: Springer Science & Business Media; 1999.

[31] Hosmer Jr DW, Lemeshow S, Sturdivant RX. Applied logistic regression. New Jersey, USA: John Wiley & Sons; 2013. p. 398.

[32] Breiman L. Random forests. Mach Learn. 2001;45:5–32.

[33] Chen T, Guestrin C. XGBoost: A scalable tree boosting system. In: KDD’16: Proceedings of the 22nd ACM SIGKDD international conference on knowledge discovery and data mining; 2016. p. 785–794.

[34] Olson DL, Delen D. Performance evaluation for predictive modeling. In: Advanced data mining techniques, Berlin, Heidelberg: Springer, 2008. p. 137–147.

[35] Han J, Pei J, Tong H. Data mining: Concepts and techniques, 4th ed. Waltham, USA: Morgan Kaufmann Publishers; 2022.

[36] Jain AK, Dubes RC. Algorithms for clustering data. New Jersey, USA: Prentice-Hall, Inc.; 1988.

[37] Page MJ, McKenzie JE, Bossuyt PM, et al. The PRISMA 2020 statement: An updated guideline for reporting systematic reviews. BMJ. 2021;372:n71. 33782057 10.1136/bmj.n71 PMC8005924

[38] Souza AB, Santos MS, Cintra MACT. Multicriteria decision analysis: Rapid review about the criteria used in the health technology assessment. J Bras Econ Saúde. 2018;10(1):64–74.

[39] Nunes LMN, Fonteles MMdF, Passos ACB, Arrais PSD. Evaluation of demands of inclusion, exclusion and alteration of technologies in the Brazilian Health System submitted to the National Committee on Technology Incorporation. Braz J Pharm Sci. 2017;53(2):1–12.

[40] Koga NM, Palotti PLM, Pinheiro JMMMS. Políticas públicas e usos de evidências no Brasil: Conceitos, métodos, contextos e práticas. Brasília: IPEA; 2022. p. 897.

