# Supplementary figures and images for: Application of natural language processing to predict final recommendation of Brazilian health technology assessment reports
Source: Int J Technol Assess Health Care. 2024 Apr 12;40(1):e19. doi: 10.1017/S0266462324000163 (PMC11569907; doi:10.1017/S0266462324000163)

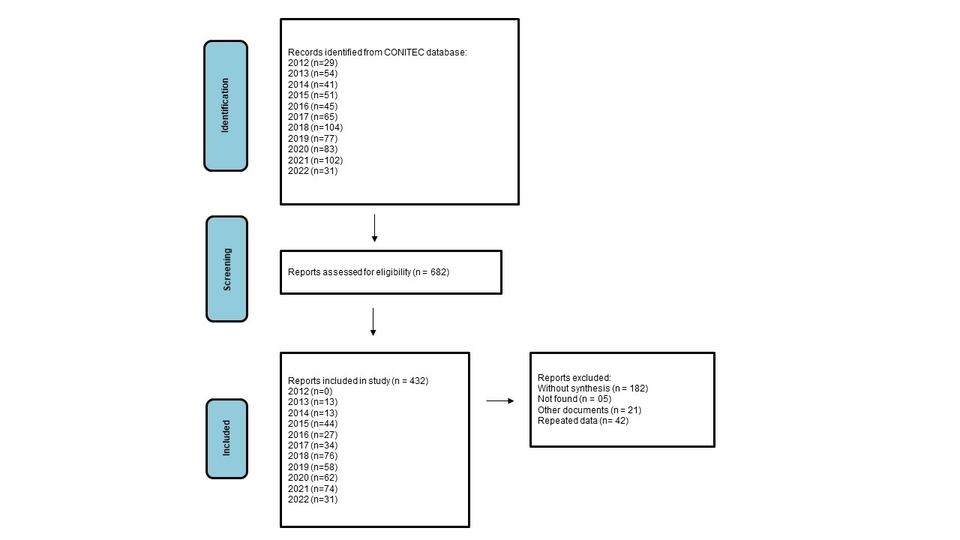

Supplement: Cardoso et al. supplementary material [file S0266462324000163sup001.jpg]
